# Supplementary material for: The relationship between weight history and psychological health—Differences related to gender and weight loss patterns
Source: PLoS One. 2023 Feb 13;18(2):e0281776. doi: 10.1371/journal.pone.0281776 (PMC9925088; doi:10.1371/journal.pone.0281776)
Supplement: Attachment — Submitted filename: Responds to Reviewer.docx [file pone.0281776.s001.docx]

Responds to Reviewer PONE-D-22-11713

The relationship between obesity, weight history and psychological health – are there differences related to gender?

Reviewer #1:

This paper describes an observational cohort study of the associations between anxiety and depression scores and obesity onset, weight loss goals and weight loss attempts in adults with obesity. The study is potentially important and valuable and the authors have a great opportunity to make a major contribution to the filed with the large amount of data they have collected. However, I have a number of comments to make about the paper, offered in a constructive spirit.

1. From the outset, there is an overwhelming sense of vagueness to the paper that pervades every part of it. The title is vague and does not adequately convey what the hypothesis is. What is the study design? What are the specific outcomes? In the abstract, the methods aren’t at all clear, the aims aren’t consistent with the title and the results just don’t make sense. Why the F statistic in the abstract results? This is unconventional.

>>> We thank the reviewer for this comment. Title and abstract haven been revised accordingly.

2. The authors need to revise STROBE (or similar) guidelines and adhere to these in revising their paper. Also it is worth noting that the formatting is not of a sufficiently high standard – taking the abstract as but one example.

>>> We agree with the reviewer and revised the manuscript with regard to the format.

3. The introduction is not adequately focussed on the hypothesis in hand. Arguably, the first two paragraphs could be deleted conmpletely and the paper would look much more succinct and focussed. The consideration of childhood obesity “tracking” into adulthood is to me totally irrelevant to this paper and just distracts from a consideration of the frankly really interesting findings that people with obesity who have a desire to lose a greater amount of weight tend to have higher levels of anxiety and depression,

>>> We apologize for this misunderstanding. The consideration of childhood obesity tracking into adulthood was added as part of the introduction, because it reflects the main focus of this paper (“weight history”) and underlines the importance of tracking BMI and other weight-related parameters as part of successuful weight managament over the life course – as suggested by others (e.g. Berry et al., 2022; Kushner et al., 2020; Ekberg et al., 2012; Ryder et al., 2019). The finding that people with obesity who have a desire to lose a greater amount of weight tend to have higher levels of anxiety and depression was not the main focus of this study. However, we think that the structure of the introduction may have caused confusing, therefore we revised this section thoughtfully.

4. Stop referring to the cohort as “non-clinical” – it diminishes the credibility of the paper.

>>> We apologize for this misunderstanding. The aim was to make clear that we did not recruit patients from a hospital or GP practice like other studies. In other words, participants in our study may or may not be under treatment at the time of the data collection. We now changed this according to the reviewers’ comment.

5. How was BMI measured by phone? Have the authors considered the challenges this poses in terms of the reliability of the data? Is tehre a precedent for large studies using self reported weight and height? If so, it would be good for the authors to demonstrate an awareness of this.

>>> We apologize for this misunderstanding. BMI was not “measured” by phone. Participants were asked to state their weight and height and BMI was then calculated from their response according to the formula provided by the WHO. In order to avoid missing values, participants who refused to provide information on weight were given a specific weight range, being able to assign them to one of the BMI categories (see Benecke, 2003 for more information). We are aware of the issues associated with self-reported weight and height, therefore we added this to the limitation section.

6. The term “weight history” is vague. So is psychological health. Be specific about what these are much earlier and throughout the paper.

>>> We thank the reviewer for this comment. “Weight history” and “psychological health” have now been described in greater detail throughout the manuscript.

7. The failure to include error bars in figures 1 and 2 is a major omission.

>>> We apologize for this mistake. Error bars have now been added to the figures.

8. The paper needs to be much shorter and more focussed and specific, especially the discussion and the introduction. Then the authors will have an important contribution to the literature.

>>> We agree with the reviewer and revised the introduction and discussion section.
